# Defensive Resistance of Cowpea *Vigna unguiculata* Control *Megalurothrips usitatus* Mediated by Jasmonic Acid or Insect Damage

**DOI:** 10.3390/plants12040942

**Published:** 2023-02-19

**Authors:** Tao Li, Mingyue Feng, Yuanming Chi, Xing Shi, Zilin Sun, Zhen Wu, Aomei Li, Wangpeng Shi

**Affiliations:** 1 Department of Entomology and MOA Key Laboratory of Pest Monitoring and Green Management, College of Plant Protection, China Agricultural University, Beijing 100193, China; 2 Plant Protection Station of Guangxi Zhuang Autonomous Region, Nanning 530007, China; 3 Shenzhen Branch, Guangdong Laboratory of Lingnan Modern Agriculture, Genome Analysis Laboratory of the Ministry of Agriculture and Rural Affairs, Agricultural Genomics Institute at Shenzhen, Chinese Academy of Agricultural Sciences, Shenzhen 518000, China; 4 Key Laboratory of Sugarcane Biotechnology and Genetic Improvement (Guangxi), Ministry of Agriculture and Rural Affairs/Guangxi Key Laboratory of Sugarcane Genetic Improvement/Sugarcane Research Institute, Guangxi Academy of Agricultural Sciences, Nanning 530007, China

**Keywords:** cowpea, bean flower thrips, transcriptome, metabolome, induced resistance

## Abstract

*Vigna unguiculata* is a vital vegetable crop in Southeast Asia, and *Megalurothrips usitatus* can cause huge damage to this crop. Enhancing the resistance of *V*. *unguiculata* against *M*. *usitatus* is a promising way to protect this crop; however, there is limited information regarding the mechanism underlying the resistance of *V*. *unguiculata* against *M*. *usitatus*. Here, a behavior assay was performed to explore the resistance of *V. unguiculata* against *M. usitatus* after insect damage or treatment by jasmonic acid (JA). Furthermore, transcriptome and metabonomics analysis was used to detect the putative mechanism underlying the resistance of *V. unguiculata* against *M. usitatus*. The pre-treatment of *Vigna unguiculata* with JA or infestation with *Megalurothrips usitatus* alleviated the damage resulting from the pest insect. We further identified differentially expressed genes and different metabolites involved in flavonoid biosynthesis and alpha-linolenic acid metabolism. Genes of chalcone reductase and shikimate O-hydroxycinnamoyltransferase involved in flavonoid biosynthesis, as well as lipoxygenase and acyl-CoA oxidase involved in alpha-linolenic acid metabolism, were upregulated in plants after herbivory or JA supplementation. The upregulation of these genes contributed to the high accumulation of metabolites involved in flavonoid biosynthesis and the alpha-linolenic acid metabolism pathway. These transcriptional and metabolite changes are potentially responsible for plant defense and a putative regulatory model is thus proposed to illustrate the cowpea defense mechanism against insect attack. Our study provides candidate targets for the breeding of varieties with resistance to insect herbivory by molecular technology.

## 1. Introduction

*Megalurothrips usitatus* (Bagrall) is a major pest worldwide and can reduce yield, causing significant economic losses each year [1]. *M. usitatus* usually destroys petals and fruits of plants, leading to severe yield failure [2]. Chemical pesticides, such as spinetoram, acetamiprid, chlorfenapyr, and fluridine nitrile, are frequently applied to control this pest; however, this rapidly causes severe resistance and environmental pollution [3]. Moreover, chemical control is usually difficult because this pest is able to hide inside the flowers and pods of plants [4]. Therefore, developing alternative approaches to control this pest is an urgent matter. Cowpea (*Vigna unguiculata* spp. sesquipedialis), which has high nutritional value, is a well-known vegetable crop that is widely cultivated in East and Southeast Asia [5,6]. In recent years, *M. usitatus* has caused serious effects on *Asparagus* bean (a subspecies of cowpea) in southern China [2].

The direct or indirect defense resistance of plants can be induced by herbivory. Direct induced resistance refers to the constitutive production of a series of defensive compounds that can affect the feeding, growth, and survival of herbivores [7]. Many studies have shown that defensive compounds are mediated by direct defense substances containing phenolics, flavonoids, and tannins [8,9]. Jasmonic acid (JA) and insect herbivory is important to eliciting plant defense against herbivory by inducing many defense responses [10,11,12], and the above-mentioned phenomena have been reported in many plants [13,14].

An increasing number of studies have shown that inducing the resistance of plants against herbivory is a promising method for pest control [15]. The combination of transcriptome and metabolomics is a classical technique to explore plant defense mechanisms, and numerous studies have been conducted [16]. Thus, in this work, association analysis was performed on the transcriptome and metabolome to identify key genes that may confer plant resistance to *M*. *usitatus*. This study will help to discover the mechanisms underlying induced resistance, which is critical for utilizing gene resources to design plants with resistance or using defensive compounds to protect plants from herbivory.

## 2. Results

### 2.1. Plant Resistance Induced by Jasmonic Acid or Insect Feeding

The insect performance preferences were identified for the three treatment groups including the healthy plant group (plants were not attacked by pest), plants sprayed with JA, and plants that were attacked after 8, 16, 24, 48, and 72 h. The insects showed an obvious preference for healthy plants (*p* < 0.05). The feeding indexes (the number of pests gathered on each leaf group/total number of insects) of healthy plants and plants at 8 and 72 h after inoculation with thrips were 0.29639805, 0.18752544, and 0.17032967, respectively; however, the areas of leaf damage were less in the plants sprayed with JA for 24 h and plants at 16, 24, and 48 h after inoculation with thrips (0.13665039, 0.07783883, 0.05006105, and 0.08119658, respectively). Therefore, it is likely that jasmonic acid or insect feeding can induce plant resistance (Table 1). As a result, 0, 24, and 48 h and JA treatments were considered as the four key treatments in our experiment. Leaf samples from CK and 24 and 48 h and JA were used for RNA-seq and metabolome analysis.

### 2.2. Profiling of Transcriptome Sequencing and Gene Expression

**Figure 1:**
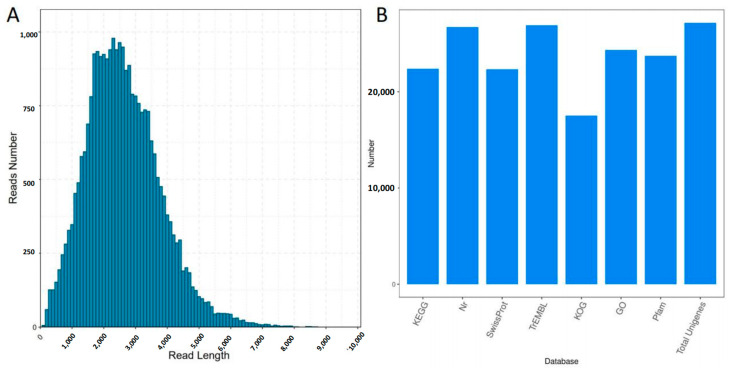
(**A**) Isoform length distribution diagram. (**B**) Transcript annotated statistical chart.

**Figure 2:**
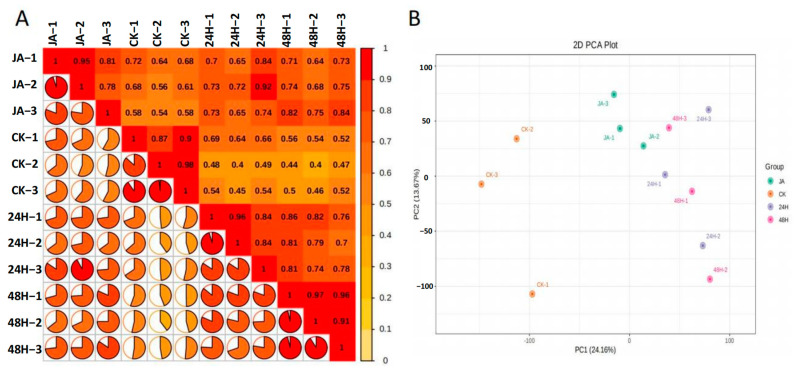
(**A**) Sample correlation diagram. (**B**) Sample PCA diagram.

To further explore the mechanisms underlying the insect resistance of plants induced by jasmonic acid and insect damage, transcriptome analysis of the leaves was performed. Because no high-quality reference genome was available, a full-length transcriptome was constructed. A total of 15,198,519 subreads were obtained with an average length of 2339 bp and an N50 of 2900 bp. After filtering out the low-quality reads (quality < 0.9 or full passes < 3), 275,887 full-length nonchimeric reads and 33,304 polished high-quality isoforms remained, and most isoforms ranged from 1000 to 5000 bp (Figure 1A). Finally, 27,167 nonredundant transcripts were obtained after removing redundant reads. By mapping these full-length transcripts to the NR, NT, Pfam, GO, and KOG databases, a total of 26,938 transcripts were annotated in at least one of these databases (Figure 1B and Table S1). Healthy leaves (CK), leaves of herbivores after 24 h (24H) and 48 h (48H), and treatment with JA (JA) in three biological replicates were used for RNA-seq analysis. Ultimately, a total of 272,618,481 reads was generated. The total mapped read rates ranged from 73.84% to 76.68% (Table S2). Correlation analysis revealed that the correlations between the three replicates were reliable (Figure 2A). In the principal component analysis (PCA), the first principal component (PC1) explained 24.16% of the variation, and the second principal component (PC2) explained 13.67% of the variation. Moreover, samples were separated by JA treatment or herbivory attack along PC1 (Figure 2B).

### 2.3. Functional Classification of Differentially Expressed Genes Based on Kyoto Encyclopedia of Genes and Genomes Analyses

**Figure 3:**
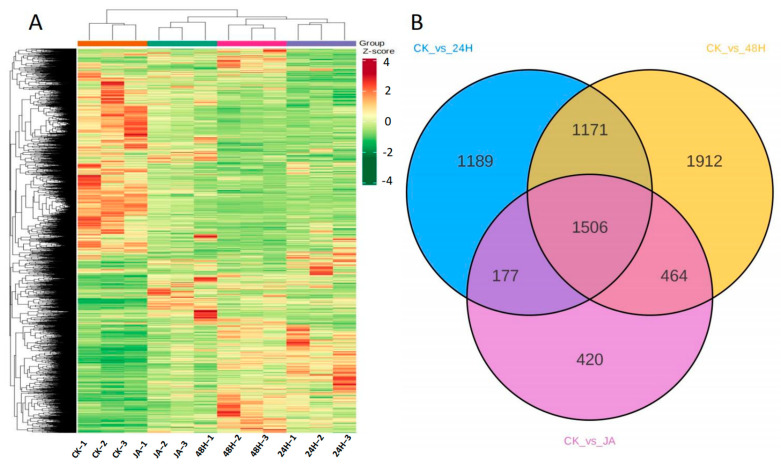
(**A**) Hierarchical cluster analysis. Different columns in the figure represent different samples, and different rows represent different genes. The colors from blue to yellow indicate increasing gene expression levels. (**B**) Venn diagram of DEGs detected in CK vs. 24H, CK vs. 48H, and CK vs. JA.

To dissect gene expression changes in plants after herbivory or being triggered by JA, differentially expressed genes (DEGs) were identified with the criteria of false discovery rate (FDR) < 0.05 and absolute value of log2ratio ≥ 1. In the comparison of CK and 24H, 4043 DEGs (Table S3) were found, with 2311 downregulated and 1732 upregulated, and the number of DEGs increased to 5053 (Table S4) (2852 downregulated and 2201 upregulated) in CK vs. 48H; however, only 2567 DEGs (Table S5) (1327 downregulated and 1240 upregulated) were found in CK vs. JA (Figure 3A). The number of upregulated DEGs was less than the number of downregulated DEGs in all the comparisons. Moreover, the number of DEGs in the herbivory group was higher than that in the JA treatment group, implying that gene expression levels changed more dramatically after insect attack than after JA treatment. A Venn diagram of DEGs was constructed to illustrate the three comparisons. A total of 1506 DEGs appeared simultaneously in all comparisons, and 1189, 1912, and 420 were unique to CK vs. 24H, CK vs. 48H, and CK vs. JA, respectively (Figure 3B and Table S6).

**Figure 4:**
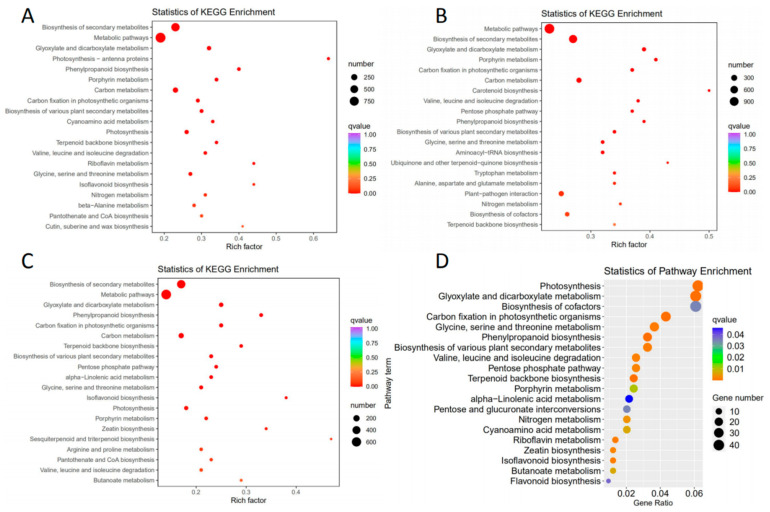
KEGG pathway enrichment analysis of DEGs. The 20 most enriched KEGG pathways. (**A**) CK vs. 24H. (**B**) CK vs. 48H. (**C**) CK vs. JA. (**D**) Venn.

Then, DEGs identified in each comparison were subjected to KEGG pathway analysis. Seventeen, sixteen, and fifteen pathways were found to be significantly enriched in CK vs. 24H (Table S7), CK vs. 48H (Table S8), and CK vs. JA (Table S9), respectively (Figure 4A–C). These pathways mainly consisted of amino acid, sugar, and secondary metabolism pathways. To more accurately confirm the common genes that occurred in all the comparison groups, KEGG analysis of common DEGs was conducted, and enriched pathways were related to phenylpropanoid biosynthesis, terpenoid backbone biosynthesis, flavonoid biosynthesis, alpha-linolenic acid metabolism, isoflavonoid biosynthesis, and biosynthesis of various plant secondary metabolites, among other pathways (Figure 4D and Table S10).

### 2.4. Induction of Flavonoid Biosynthesis, Phenylpropanoid Biosynthesis and Alpha-Linolenic Acid Metabolism by Insect Feeding or Exogenous JA

Genes involved in flavonoid biosynthesis and alpha-linolenic acid metabolism have been proven to be responsible for resistance induced in plants (Table 2). Therefore, we further focused on DEGs associated with these pathways. Among the 24 DEGs involved in the phenylpropanoid biosynthesis pathway, most DEGs were upregulated, with seventeen and seven genes being up- and downregulated, respectively. The downregulated genes were two peroxidases [EC:1.11.1.7], four cinnamoyl-CoA reductases [EC:1.2.1.44], and one shikimate O-hydroxycinnamoyltransferase [EC:2.3.1.133], and upregulated genes included seven peroxidases [EC:1.11.1.7], one 4-coumarate--CoA ligase [EC:6.2.1.12], four cinnamoyl-CoA reductases [EC:1.2.1.44], two coniferyl-alcohol glucosyltransferases [EC:2.4.1.111], one shikimate O-hydroxycinnamoyltransferase [EC:2.3.1.133], and two cinnamyl-alcohol dehydrogenases [EC:1.1.1.195].

When focused on flavonoid biosynthesis-related genes, seven DEGs were identified, and almost all the DEGs were induced under feeding or JA, except shikimate O-hydroxycinnamoyltransferase, which was downregulated, and two chalcone isomerases [EC:5.5.1.6], three chalcone reductases, and one shikimate O-hydroxycinnamoyltransferase [EC:2.3.1.133], which were upregulated. Most DEGs associated with alpha-linolenic acid metabolism were induced, including one phospholipase A1 [EC:3.1.1.32], one alcohol dehydrogenase class-P [EC:1.1.1.1], four phospholipase A1 genes, one fatty acid alpha-dioxygenase [EC:1.13.11.92], one acetyl-CoA acyltransferase 1 [EC:2.3.1.16], two hydroperoxide dehydratases [EC:4.2.1.92] | (RefSeq) allene oxide synthase 1, two lipoxygenases [EC:1.13.11.12], and two acyl-CoA oxidases [EC:1.3.3.6].

### 2.5. Dynamic Transcriptome Responses to Insect Attack

**Figure 5:**
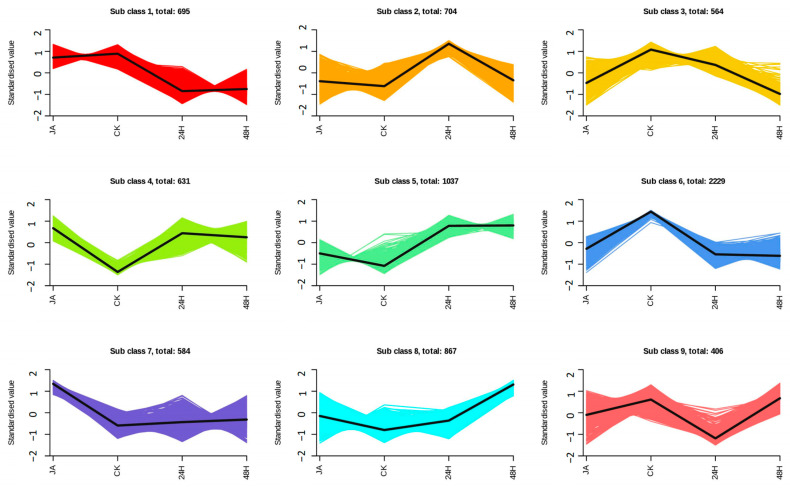
K-MEAN analysis of transcripts in different samples; the *y*-axis is the standardized value, which represents the expression level of each cluster in different groups.

From the K-MEAN analysis results, nine clusters representing 7717 genes are shown in Figure 5 (Table S11). Subclass 6 contained the most transcripts (2229), and subclass 9 contained only 406 transcripts. A total of 600 transcription factors belonging to 72 families were identified. In subclusters 2, 4, and 5, we found that the expression patterns in the treatment group were higher than those in the control group. However, clusters 3, 6, and 9 showed adverse trends. Among the TFs that occurred in subclusters 2, 3, 4, 5, 6, and 9, MYB (13), AP2/ERF-ERF (19), WRKY (14), and bZIP (12) were included. These TFs exhibit different treatment-specific expression patterns, and the specific expression patterns of these may reflect pivotal roles in plant defense.

### 2.6. Metabolite Profiles Indicate Differences in Metabolic Regulation under Herbivory Attack and JA Treatment

**Figure 6:**
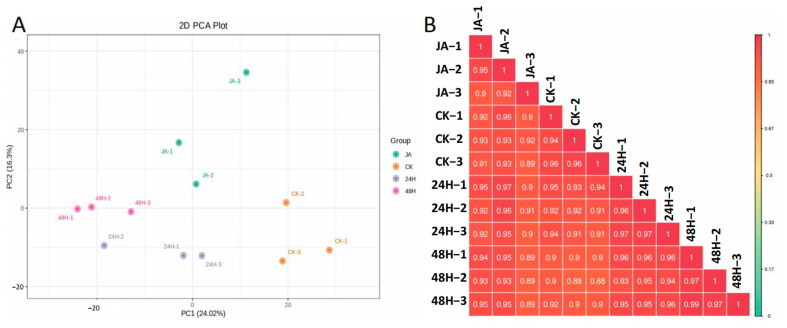
(**A**) PCA of primary metabolites from plants grown under control or induced conditions. Scores of Principle Component (PC) 1 are plotted against PC2. (**B**) Pearson’s correlation coefficients of metabolites in plants under different conditions.

To further explore metabolite changes in plants, metabolomic profiles were generated for the control and treated plants. A total of 1224 metabolites were detected, and they were divided into 10 classes, among which flavonoids, phenolic acids, and lipids were main classes. Moreover, alkaloids, amino acids and derivatives, lignans and coumarins, nucleotides and derivatives, organic acids, phenolic acids, and terpenoids were also detected. The correlation analysis indicated good reproducibility of the different biologically replicated samples (Figure 6B). In PCA, PC1 and PC2 explained 24.02% and 16.3% of the variation, respectively (Figure 6A). PC1 separated the samples by herbivory attack or JA treatment. By analyzing the differentially expressed metabolites (DEMs), we found 259, 310, and 220 different metabolites in CK vs. 24H, CK vs. 48H, and CK vs. JA, respectively (Table 3 and Tables S12–S14). In the 24H group vs. CK, 24 metabolites were downregulated, and 205 were upregulated. In the comparison of the 48H group vs. CK, 66 and 244 were down- or upregulated, respectively. Moreover, in the comparison of the JA and CK groups, 62 were downregulated and 158 were upregulated. The number of DEMs in CK vs. JA was less than those in CK vs. 24H and CK vs. 48H, which were similar to the number of DEGs in the transcriptome analysis. A total of 103 different metabolites were found to overlap in all three groups. The KEGG analysis showed that these different metabolites could be attributed to secondary and primary metabolite-related pathways, including flavonoid biosynthesis, alpha-linolenic acid metabolism, phenylpropanoid biosynthesis, and alanine, aspartate, and glutamate metabolism.

For better understanding of the changes in flavonoid and alpha-linolenic acid-related metabolites after herbivory or JA induction (Table 4), we identified the DEMs associated with flavonoid biosynthesis, alpha-linolenic acid metabolism, and phenylpropanoid biosynthesis, and found 13 DEMs. Metabolites associated with flavonoid biosynthesis included trans-5-O-(p-coumaroyl) shikimate, 3,5,7-trihydroxyflavanone (pinobanksin), dihydrokaempferol, naringenin chalcone, 5-O-p-coumaroylquinic acid*, liquiritigenin, naringenin, and isoliquiritigenin. DEMs involved in alpha-linolenic acid metabolism included 9-hydroperoxy-10E,12,15Z-octadecatrienoic acid, 9-hydroxy-10,12,15-octadecatrienoic acid, 12-oxo-phytodienoic acid, 13S-hydroxy-9Z,11E,15Z-octadecatrienoic acid, and jasmonic acid. All of these metabolites were induced after herbivory or JA treatment.

### 2.7. Correlation between Flavonoid Biosynthesis, Phenylpropanoid Biosynthesis, and Alpha-Linolenic Acid Metabolism-Related Transcripts and Metabolites

**Figure 7:**
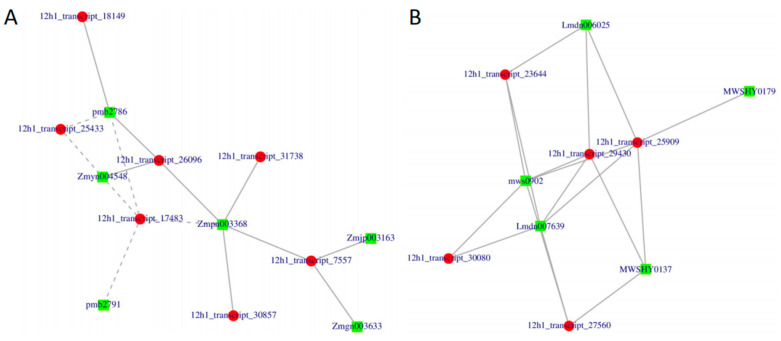
Correlation network of the DEGs and DEMs. Red represents transcripts, and green represents metabolites. (**A**) Flavonoid biosynthesis. (**B**) alpha-Linolenic acid metabolism.

To identify genes and metabolites with statistically significant correlations, the transcriptomes and metabolomes were integratively analyzed with the Pearson correlation coefficient method. Because JA and plant secondary metabolites are important compounds in defending against herbivory attack, we focused on flavonoid biosynthesis and alpha-linolenic acid metabolism. The correlation analysis revealed twenty-three significant correlations (*p* < 0.05) between six metabolites and seven transcripts in flavonoid biosynthesis (red represents transcripts, and green represents metabolites in Figure 7A). The metabolites included trans-5-O-(p-coumaroyl) shikimate, pinobanksin, dihydrokaempferol, naringenin chalcone, liquiritigenin, naringenin, and isoliquiritigenin. The transcripts included chalcone isomerase [EC:5.5.1.6] (12h1_transcript_15683; 12h1_transcript_31775), chalcone reductase (12h1_transcript_29690; 12h1_transcript_30450), and shikimate O-hydroxycinnamoyltransferase [EC:2.3.1.133] (12h1_transcript_27132; 12h1_transcript_26689). In alpha-linolenic acid metabolism, the correlation analysis revealed significant correlations (*p* < 0.05) between five metabolites and five transcripts (red represents transcripts, and green represents metabolites in Figure 7B). The transcripts included phospholipase A1 (12h1_transcript_25433; 12h1_transcript_31738), lipoxygenase [EC:1.13.11.12] (12h1_transcript_30857), enoyl-CoA hydratase/3-hydroxyacyl-CoA dehydrogenase (MFP2) (12h1_transcript_17483), and acyl-CoA oxidase [EC:1.3.3.6] ACX (12h1_transcript_18149). The metabolites included 9-hydroxy-10,12,15-octadecatrienoic acid (pmb2786) [9-HOT], 9-hydroxy-10E,12,15Z-octadecatrienoic acid (pmb2791) (9-HPOT), 13S-hydroxy-9Z,11E,15Z-octadecatrienoic acid (Zmpn003368), and 12-oxo-phytodienoic acid (Zmyn004548) (OPDA).

## 3. Discussion

JA and insect feeding were reported to play vital roles in plant-induced defense against herbivores because they can reduce insect damage [11,12]. This may be caused by the accumulation of defensive compounds induced by insect feeding or JA application. In our study, less damage was observed in plants pretreated by insect herbivory or JA than in healthy leaves. Drastic changes in the transcriptome and metabolome also occurred. In the group comparisons, 4043, 5053, and 2567 DEGs were identified in CK vs. 24H, CK vs. 48H, and CK vs. JA, respectively, and the metabolome analysis showed that there were 259, 310, and 220 different metabolites in CK vs. 24H, CK vs. 48H, and CK vs. JA, respectively. Generally, the insect-induced resistance of plant depends on producing a series of defensive compounds to affect the feeding, growth, and survival of herbivores [7,8,9]. In addition, the defense response of plants induced by JA or insect attack is mediated by these compounds in the phenylpropanoid biosynthesis pathway, flavonoid biosynthesis, and alpha-linolenic acid metabolism, which have been identified in many plants [13,14]. In our study, seven peroxidases [EC:1.11.1.7] involved in the phenylpropanoid biosynthesis pathway were found to be upregulated, which might play an important role in mediating the defensive responses of cowpea to insect damage [17]. Moreover, four cinnamoyl-CoA reductases [EC:1.2.1.44], one shikimate O-hydroxycinnamoyl transferase [EC:2.3.1.133], and two cinnamyl-alcohol dehydrogenases [EC:1.1.1.195] involved in this pathway were also induced by insect attack or JA; these genes are known to perform critical functions under biotic stress condition [18]. In addition, two chalcone isomerases [EC:5.5.1.6], three chalcone reductases, and one shikimate O-hydroxycinnamoyltransferase [EC:2.3.1.133] were upregulated. These genes were associated with flavonoid biosynthesis. Flavonoids are the secondary compounds that play roles in plant defenses, which might be the result of changes in some metabolites that lead to the alleviation of insect damage. A similar study indicated that JA alleviates pest-associated losses in groundnut by mediating POD, PPO, and other defensive compounds, such as phenols, H_2_O_2_, and MDA (malondialdehyde) [19]. Plants protect themselves from pest attack with a large number of secondary metabolites [20]. These metabolites usually inhibit insect damage because they are feeding deterrents [21]. Plant metabolites may manipulate feeding behavior of herbivores [22]. Metabolites associated with flavonoid biosynthesis included trans-5-O-(p-coumaroyl) shikimate, 3,5,7-trihydroxyflavanone (pinobanksin), dihydrokaempferol, naringenin chalcone, 5-O-p-coumaroylquinic acid, liquiritigenin, naringenin, and isoliquiritigenin, which were herbivory- or JA-induced and accumulated in the cowpeas. Naringenin plays key roles in inhibiting the growth and development of the common cutworm *Spodoptera litura* [23]. The growth rate of tea geometrids showed significant decreases by feeding an artificial diet supplemented with naringenin [24]. In this study, seven metabolites and six transcripts in flavonoid biosynthesis were significantly correlated. The metabolites included trans-5-O-(p-coumaroyl) shikimate, pinobanksin, dihydrokaempferol, naringenin chalcone, liquiritigenin, naringenin, and isoliquiritigenin. The transcripts included chalcone isomerase [EC:5.5.1.6] (12h1_transcript_15683; 12h1_transcript_31775), chalcone reductase (12 h1_transcript_29690; 12h1_transcript_30450), and shikimate O-hydroxycinnamoyltransferase [EC:2.3.1.133] (12h1_transcript_27132; 12h1_transcript_26689). Therefore, we assumed that DEGs would lead to a difference in secondary metabolite accumulation in cowpea, and then modulate the feeding behavior of thrips. A previous study confirmed that secondary metabolites are critical in modulating feeding behavior of insects [25]. The defensive roles of jasmonic acid and flavonoid pathways have been widely reported in a large range of plants [22].

JA is a well-known phytohormone of plant defense, and alpha-linolenic acid metabolism could produce precursors for JA biosynthesis [22]. In our study, several genes related to alpha-linolenic acid metabolism were upregulated in cowpea after herbivory or JA treatment, including two lipoxygenases [EC:1.13.11.12]. Lipoxygenases (LOXs) are vital antioxidative enzymes associated with plant defense [26]. Induction of LOX activity has been reported in many plants after attack by herbivory [27]. Therefore, this gene is vital in plant defense. Moreover, many alpha-linolenic acid metabolism-related metabolites accumulated after induction by herbivory and JA, such as 9-hydroxy-10E,12,15Z-octadecatrienoic acid, 9-hydroxy-10,12,15-octadecatrienoic acid, 12-oxo-phytodienoic acid, 13S-hydroxy-9Z,11E,15Z-octadecatrienoic acid, and jasmonic acid. The associations of the transcriptome and metabolome revealed that the differential expression of phospholipase A1 (12h1_transcript_25433; 12h1_transcript_31738), lipoxygenase [EC:1.13.11.12] (12h1_transcript_30857), 13S-hydroxy-9Z,11E,15Z-octadecatrienoic acid (12h1_transcript_30857), enoyl-CoA hydratase/3-hydroxyacyl-CoA dehydrogenase (MFP2) (12h1_transcript_17483), and acyl-CoA oxidase [EC:1.3.3.6] ACX (12h1_transcript_18149) might lead to the accumulated changes in fluctuations of these metabolites, including 9-hydroxy-10,12,15-octadecatrienoic acid (pmb2786) [9-HOT], 9-hydroxy-10E,12,15Z-octadecatrienoic acid (pmb2791) (9-HPOT), 13S-hydroxy-9Z,11E,15Z-octadecatrienoic acid (Zmpn003368), and 12-oxo-phytodienoic acid (Zmyn004548) (OPDA). OPDA plays a role in regulating plant defense against nematodes in Arabidopsis [28]. 12-oxo-Phytodienoic acid (OPDA) is a primary precursor of (-)-jasmonic acid (JA) and is responsible for the defenses of many plants. Jasmonates are derived from oxygenized FAs, linolenic acid (18:3), and hexadecatrienoic acid (16:3). During this biosynthesis process, the conversion of hydroperoxide to 13(S)-hydroperoxy-octadecatrienoic acid is catalyzed by lipoxygenases [29,30]. These pathways have been activated in plants suffering from abiotic or biotic stress, including insect attack [31,32]. A previous study indicated that elevated acyl-CoA oxidase (ACX) activity leads to strong plant resistance to necrotrophic *Botrytis cinerea*, whereas the ACX mutant showed enhanced susceptibility to *B. cinerea* due to the reduced JA level [33]. Therefore, the defensive role of jasmonic acid-related pathways was critical for the induced resistance in this study.

Transcription factors are responsible for many biological processes in plants, including development, growth, and defense [34,35,36]. In our study, K-means clustering revealed that MYB (13), AP2/ERF-ERF (19), WRKY (14), and bZIP (12) showed different expression patterns in the pretreated plants, and CmMYB19, WRKY7, WRKY58, WRKY62, WRKY64, and WRKY76 were found to be induced under pathogen or insect attack [37,38]. The induction of bZIP TFs was observed to contribute to the defense response against greenbug infestation in sorghum [39]. Therefore, these transcription factors have a potential role in mediating plant defense.

**Figure 8:**
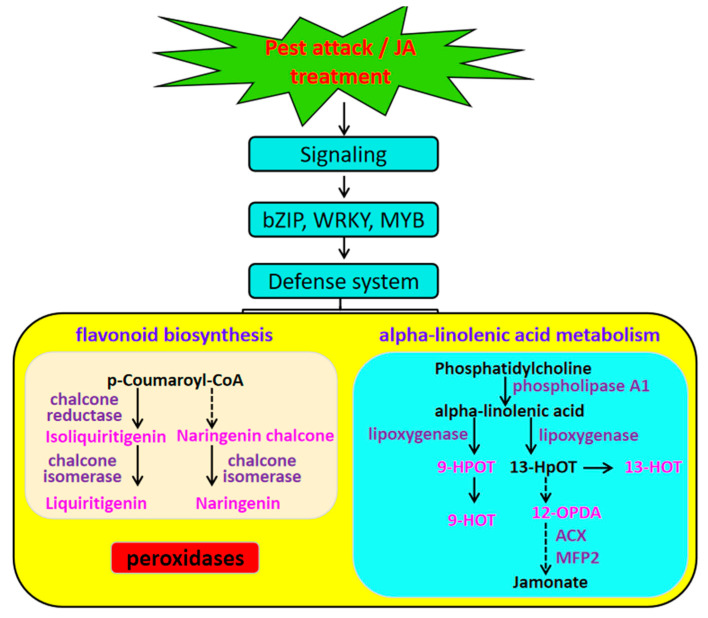
Putative model of cowpea’s response to *Megalurothrips usitatus* herbivory. The names in red are metabolite compounds. The DEGs are exhibited in purple below the arrows. 9-HPOT: 9-hydroxy-10E,12,15Z-octadecatrienoic acid; 9-HOT: 9-hydroxy-10,12,15-octadecatrienoic acid; 13-HOT: 13S-hydroxy-9Z,11E,15Z-octadecatrienoic acid; 12-OPDA: 12-oxo-phytodienoic acid; ACX: acyl-CoA oxidase; MFP2: enoyl-CoA hydratase/3-hydroxyacyl-CoA dehydrogenase. WRKY and MYB were the transcription factors identified in our study; however, their interaction with the downstream gene was unclear. The signaling is the common complex path triggered by the external stimulus.

In summary, the present study revealed that the pretreatment of *Vigna unguiculata* with JA or infestation with *Megalurothrips usitatus* contributed to induced insect resistance of host plants. The mechanism of insect resistance was associated with changes in secondary metabolites or phytohormone precursors at the transcriptome and metabolome levels, including chalcone reductase, chalcone isomerase, and lipoxygenase. Moreover, the defense mechanism involved a number of TFs such as MYB, WRKY, and bZIP (Figure 8). Using molecular technology, the study provided candidate targets for the breeding of varieties with resistance to insect herbivory.

## 4. Materials and Methods

### 4.1. Plant Material, Cultivation, and Experimental Design of Megalurothrips usitatus Herbivory Treatment

The colony of *M. usitatus* used in this study was reared in the laboratory as described previously [3]. The cowpea *V. unguiculata* was planted in an artificial climate box with the relative humidity being 75%. Insects were reared on the cultivated cowpea in a growth chamber (26 ± 1 °C, 65 ± 5% relative humidity, 16:8 light/dark photoperiod). Plant materials were divided into three groups. The first group was infected with insects which were placed on the surface of plant leaves for 8, 16, 24, 48, and 72 h, respectively (including 4 treatments). The second group was sprayed with JA (including 1 treatment) and healthy plant leaves were used as the control group (including 1 treatment). The above three groups were then attacked by thrips. There were 6 treatments, namely, healthy control (CK), and 8, 16, 24, 48 and 72 h in this study. The leaves of these 6 treatments were used for the herbivory preference, and then the tested insect was used to choose the leaves from the 6 treatments. This process persisted for 48 h, and after that, the number of insects gathered on each specific treatment group was recorded, and the insect used in the choice experiment was also documented. The numbers of pests gathered on each leaf group/total number of insects were calculated using a stereoscope. A total of 20 *M. usitatus* for the insect damage was used in the experiment with three biological repetitions. Before being used, all the insects were not supplied with food for 24 h, and adult insects were used in this study.

### 4.2. Gene Expression Analysis Using RNA-seq

Total RNA was extracted and purified using the Plant RNA Kit (BioTeke, Beijing, China) according to the manufacturer’s instructions. The Agilent 2100 bioanalyzer was applied to assess the RNA integrity. The mRNA was enriched by magnetic beads with Oligo (dT) and the first strand of cDNA was synthesized using the fragmented mRNA as the template. Then, the RNA strand was degraded by RNaseH and the second strand of cDNA was synthesized using the DNA Polymerase I system. Subsequently, the cDNA library purity, quantity, and integrity were assessed using a Qubit 2.0 Fluorometer and Agilent 2100 Bioanalyzer prior to sequencing by an Illumina NovaSeq 6000 (Illumina Inc., San Diego, CA, USA).

Iso-Seq library construction was performed according to the Isoform Sequencing protocol (Iso-Seq) and the Clontech SMARTer PCR cDNA Synthesis Kit protocol (Mountain View, CA, USA). The sequencing data were processed using the SMRTlink software (Menlo Park, CA, USA). Briefly, subread BAM files were converted to circular consensus sequences (CCSs). Full-length nonchimeric (FLNC) sequences were selected from the CCS and then were subjected to nonredundant and cluster treatment by the ICE Quiver algorithm and Arrow polishing with nFL sequence. Finally, high-quality and polished full-length consensus sequences were produced [40,41].

Diamond blastx was used to perform gene function annotation with an E-value < 10^−5^ [42]. The annotation datasets were taken from the NCBI nonredundant (NR) protein sequences, Swiss-Prot, Trembl, KEGG, GO, and KOG/COG databases. Transcripts were then aligned to the PFAM (Protein family) database to perform annotation using Hmmscan. Gene expression levels were calculated using RSEM [43]. FPKM was used to estimate gene expression levels. DEGs were analyzed using DESeq2 with cutoff padj < 0.05 and fold change > 2 [44]. GOseq and KOBAS were used for GO and KEGG enrichment analyses, respectively. The results of TF analysis of differentially expressed genes were extracted directly from the AnimalTFDB database [45].

### 4.3. Metabolite Profiling and Data Analysis

Biological samples were freeze-dried using a vacuum freeze-dryer (Scientz-100F, Anqing, China). Each freeze-dried sample was crushed using a mixer mill (MM 400, Retsch, Haan, Germany) with zirconia beads for 1.5 min at 30 Hz. Fifty milligrams of lyophilized powder were dissolved in 1.2 mL 70% methanol solution and vortexed for 30 s every 30 min for a total of 6 times. Following centrifugation at 12,000 rpm for 3 min, the extracts were filtered (SCAA-104, 0.22 µm pore size; ANPEL, Shanghai, China, http://www.anpel.com.cn/, access on 16 December 2022) before UPLC–MS/MS analysis.

Unsupervised PCA (principal component analysis) was performed by the statistics function prcomp within R (www.r-project.org, access on 16 December 2022). The Pearson correlation coefficients (PCCs) between samples were calculated by the cor function in R and presented as only heatmaps. For two-group analyses, differential metabolites were determined by VIP (VIP ≥ 1) and absolute Log2FC (|Log2FC| ≥ 1.0). Identified metabolites were annotated using the KEGG Compound database (http://www.kegg.jp/kegg/compound/, access on 16 December 2022), and annotated metabolites were then mapped to the KEGG Pathway database (http://www.kegg.jp/kegg/pathway.html, access on 16 December 2022). Pathways with significantly regulated metabolites mapped to them were then fed into MSEA (metabolite set enrichment analysis), and their significance was determined by hypergeometric test *p* values.

### 4.4. Metabolomic and Transcriptomic Association Analysis

Pearson correlation coefficients were used to analyze the correlations between the differentially expressed genes from transcriptome analysis and the differential metabolites from metabolome analysis. If the correlation coefficient was less than 0, the correlation was negative, and vice versa. The correlated pairs with *p* values < 0.05 were used for further analysis. The differential genes and metabolites obtained were mapped to the KEGG pathway database simultaneously to obtain their common pathway information.

## Figures and Tables

**Table 1:** Summary of insect feeding performance on plants. The statistical approach was the LSD test. The herbivory insect is *M*. *usitatus*, and the plant is *V. unguita*; 20 insects were placed on the surface of the leaf in each treatment group. The feeding index means the number of pest gathered on each leaf group/total number of insects.

Index	Meaning	Value (Feeding Index)	std	Groups
CK	Healthy plants	0.29639805	0.113775679	A
8H	Plants (herbivory for 8 h)	0.18752544	0.178979875	Ab
16H	Plants (herbivory for 16 h)	0.07783883	0.02788157	B
24H	Plants (herbivory for 24 h)	0.05006105	0.05132564	B
48H	Plants (herbivory for 48 h)	0.08119658	0.083415476	B
72H	Plants (herbivory for 72 h)	0.17032967	0.018587713	Ab
JA	Plants treated with jasmonic acid	0.13665039	0.007578163	B

**Table 2:** Overlapping differentially expressed genes related to flavonoid biosynthesis, alpha-linolenic acid metabolism, and phenylpropanoid biosynthesis detected in different groups.

KEGG Pathway	ID	CK vs. JA	CK vs. 24H	CK vs. 48H	KEGG Annotation
ko00592: alpha- Linolenic acid metabolism	12h1_transcript_31738	3.87	3.21	3.40	K16818 phospholipase A1 [EC:3.1.1.32]
	12h1_transcript_27064	1.78	2.27	2.10	K00232 acyl-CoA oxidase [EC:1.3.3.6]
	12h1_transcript_20570	4.83	4.69	5.19	K10529 fatty acid alpha-dioxygenase [EC:1.13.11.92]
	12h1_transcript_17483	−1.55	−2.31	−2.53	K10527 enoyl-CoA hydratase/3-hydroxyacyl-CoA dehydrogenase [EC:4.2.1.17 1.1.1.35 1.1.1.211]
	12h1_transcript_25433	−1.06	−1.57	−2.08	K16818 phospholipase A1 [EC:3.1.1.32]
	12h1_transcript_28360	5.32	4.87	4.07	K16818 phospholipase A1 [EC:3.1.1.32]
	12h1_transcript_26243	1.22	2.02	1.83	K07513 acetyl-CoA acyltransferase 1 [EC:2.3.1.16]
	12h1_transcript_23465	11.47	10.72	11.97	K22389 phospholipase A1 [EC:3.1.1.32]
	12h1_transcript_18149	1.23	1.55	1.82	K00232 acyl-CoA oxidase [EC:1.3.3.6]
	12h1_transcript_23927	3.53	2.41	2.77	K16818 phospholipase A1 [EC:3.1.1.32]
	12h1_transcript_32755	6.65	6.94	7.31	K18857 alcohol dehydrogenase class-P [EC:1.1.1.1]
	12h1_transcript_22757	4.09	2.34	3.08	K16818 phospholipase A1 [EC:3.1.1.32]
	12h1_transcript_27229	2.37	1.08	1.21	K01723 hydroperoxide dehydratase [EC:4.2.1.92]
	12h1_transcript_25528	12.24	10.98	8.70	K01723 hydroperoxide dehydratase [EC:4.2.1.92]
	12h1_transcript_30857	2.15	1.45	1.85	K00454 lipoxygenase [EC:1.13.11.12]
	12h1_transcript_11874	2.32	1.15	1.19	K00454 lipoxygenase [EC:1.13.11.12]
Flavonoid biosynthesis	12h1_transcript_31775	2.03	2.41	2.34	K01859 chalcone isomerase [EC:5.5.1.6]
	12h1_transcript_27132	−3.09	−3.44	−4.34	K13065 shikimate O-hydroxycinnamoyltransferase [EC:2.3.1.133]
	12h1_transcript_29690	1.06	1.68	2.35	K08243 chalcone reductase
	12h1_transcript_15191	3.67	3.74	4.06	K08243 chalcone reductase
	12h1_transcript_30450	2.67	3.03	3.00	K08243 chalcone reductase
	12h1_transcript_26689	1.13	1.45	1.54	K13065 shikimate O-hydroxycinnamoyltransferase [EC:2.3.1.133]
	12h1_transcript_15683	1.58	1.56	1.12	K01859 chalcone isomerase [EC:5.5.1.6]
Phenylpropanoid biosynthesis	12h1_transcript_29158	5.10	5.77	5.28	K00430 peroxidase [EC:1.11.1.7]
	12h1_transcript_11139	3.93	4.14	3.53	K01904 4-coumarate--CoA ligase [EC:6.2.1.12]
	12h1_transcript_25562	−1.29	−2.34	−1.38	K09753 cinnamoyl-CoA reductase [EC:1.2.1.44]
	12h1_transcript_27132	−3.09	−3.44	−4.34	K13065 shikimate O-hydroxycinnamoyltransferase [EC:2.3.1.133]
	12h1_transcript_30559	2.73	4.05	2.95	K09753 cinnamoyl-CoA reductase [EC:1.2.1.44]
	12h1_transcript_29457	2.42	3.97	3.16	K00430 peroxidase [EC:1.11.1.7]
	12h1_transcript_28547	3.10	2.59	2.31	K00430 peroxidase [EC:1.11.1.7]
	12h1_transcript_13877	1.74	2.12	2.33	K00430 peroxidase [EC:1.11.1.7]
	12h1_transcript_24102	3.90	4.44	4.45	K22395 cinnamyl-alcohol dehydrogenase [EC:1.1.1.195]
	12h1_transcript_29639	4.53	5.53	6.59	K00430 peroxidase [EC:1.11.1.7]
	12h1_transcript_29578	1.54	2.11	2.36	K09753 cinnamoyl-CoA reductase [EC:1.2.1.44]
	12h1_transcript_29580	2.21	3.71	2.46	K09753 cinnamoyl-CoA reductase [EC:1.2.1.44]
	12h1_transcript_29601	−1.82	−1.74	−2.53	K00430 peroxidase [EC:1.11.1.7]
	12h1_transcript_24368	2.31	2.38	2.35	K22395 cinnamyl-alcohol dehydrogenase [EC:1.1.1.195]
	12h1_transcript_23722	2.95	4.50	4.02	K12356 coniferyl-alcohol glucosyltransferase [EC:2.4.1.111]
	12h1_transcript_29726	−1.15	−1.94	−2.74	K00430 peroxidase [EC:1.11.1.7]
	12h1_transcript_29085	−1.39	−2.10	−3.14	K09753 cinnamoyl-CoA reductase [EC:1.2.1.44]
	12h1_transcript_32533	3.55	5.28	3.77	K09753 cinnamoyl-CoA reductase [EC:1.2.1.44]
	12h1_transcript_17047	2.83	2.50	1.81	K00430 peroxidase [EC:1.11.1.7]
	12h1_transcript_26689	1.13	1.45	1.54	K13065 shikimate O-hydroxycinnamoyltransferase [EC:2.3.1.133]
	12h1_transcript_6047	1.02	1.23	1.22	K00430 peroxidase [EC:1.11.1.7]
	12h1_transcript_25606	3.83	3.84	4.69	K12356 coniferyl-alcohol glucosyltransferase [EC:2.4.1.111]
	12h1_transcript_26567	−1.78	−1.30	−1.80	K09753 cinnamoyl-CoA reductase [EC:1.2.1.44]
	12h1_transcript_21954	−1.16	−1.36	−2.53	K09753 cinnamoyl-CoA reductase [EC:1.2.1.44]

**Table 3:** Summary of differentially expressed metabolites in different comparisons.

Group Name	All Sig Diff	Downregulated	Upregulated
CK_vs_24H	259	54	205
CK_vs_48H	310	66	244
CK_vs_JA	220	62	158

**Table 4:** Overlapping differentially expressed metabolites related to flavonoid biosynthesis and alpha-linolenic acid metabolism detected in different groups.

KEGG PATHWAY	Index	Compounds	CK_vs_24H		CK_vs_48H		CK_vs_JA	
			*p* Value	Fold_Change	*p* Value	Fold_Change	*p* Value	Fold_Change
Flavonoid	pmb0751	Trans-5-O-(p-Coumaroyl) shikimate	0	6.8	0.13	4.65	0	4.95
	mws0914	3,5,7-Trihydroxyflavanone (Pinobanksin)	0.02	60	0.07	17.8	0.09	6.43
	mws1094	Aromadendrin (Dihydrokaempferol)	0.05	17.3	0.05	11	0.36	10.2
	pme2960	Naringenin chalcone; 2′,4,4′,6′-Tetrahydroxychalcone	0.03	44.2	0.04	16.1	0.01	6.61
	pmb3074	5-O-p-Coumaroylquinic acid *	0.02	2.65	0.09	2.44	0.03	2.32
	mws0902	Liquiritigenin	0.02	18.1	0.05	13.8	0.26	4.18
	MWSHY0137	Naringenin (5,7,4′-Trihydroxyflavanone)	0.04	35.9	0.04	11.8	0.06	5.26
	pme3217	Isoliquiritigenin	0.03	25.1	0.06	17.5	0.09	3.63
alpha-Linolenic acid metabolism	pmb2791	9-Hydroperoxy-10E,12,15Z-octadecatrienoic acid	0.19	3.16	0.02	5.2	0.11	3.44
	pmb2786	9-Hydroxy-10,12,15-octadecatrienoic acid	0.05	3.08	0.04	7.27	0.07	4.55
	Zmyn004548	12-Oxo-phytodienoic acid	0.1	3.28	0.02	7.01	0.03	5.68
	Zmpn003368	13S-Hydroxy-9Z,11E,15Z-octadecatrienoic acid	0.07	2.93	0	5.04	0.02	5.36
	Zmjp003163	Jasmonic acid	0.38	6.22	0.09	6.44	0.01	5.56

## Data Availability

The raw RNA-Seq data of this study were deposited in the Genome Sequence Archive at the China National Center for Bioinformation (https://www.cncb.ac.cn/) under project number PRJCA013915 (access on 16 December 2022).

## Supplementary Materials

The following supporting information can be downloaded at: https://www.mdpi.com/article/10.3390/plants12040942/s1, Table S1: Function annotation of unigenes in different annotation databases; Table S2: Assemble state information of unigenes; Table S3: DEGs in CK_vs_24H; Table S4: DEGs in CK_vs_48H; Table S5: DEGs in CK_vs_JA; Table S6: Venn of DEGs; Table S7: KEGG enrichment pathway of DEGs in CK_vs_24H; Table S8: KEGG enrichment pathway of DEGs in CK_vs_48H; Table S9: KEGG enrichment pathway of DEGs in CK_vs_JA; Table S10: KEGG enrichment pathway of DEGs in Venn of DEGs; Table S11: Detail information of K-means analysis; Table S12: KEGG enrichment pathway of DEMs in CK_vs_24H; Table S13: KEGG enrichment pathway of DEMs in CK_vs_48H; Table S14: KEGG enrichment pathway of DEMs in CK_vs_JA.
